# Rapid risk stratification of acute coronary syndrome: adoption of an adapted European Society of Cardiology 0/1-hour troponin algorithm in a real-world setting

**DOI:** 10.1093/ehjopen/oeac048

**Published:** 2022-07-29

**Authors:** Liam S Couch, Aish Sinha, Roshan Navin, Laura Hunter, Divaka Perera, Michael S Marber, Thomas E Kaier

**Affiliations:** King’s College London BHF Centre, The Rayne Institute, St Thomas’ Hospital, Westminster Bridge Road, London SE1 7EH, UK; King’s College London BHF Centre, The Rayne Institute, St Thomas’ Hospital, Westminster Bridge Road, London SE1 7EH, UK; Acute Medicine Department, St Thomas’ Hospital, Westminster Bridge Rd, London, UK; Emergency Department, St Thomas’ Hospital, Westminster Bridge Rd, London, UK; King’s College London BHF Centre, The Rayne Institute, St Thomas’ Hospital, Westminster Bridge Road, London SE1 7EH, UK; King’s College London BHF Centre, The Rayne Institute, St Thomas’ Hospital, Westminster Bridge Road, London SE1 7EH, UK; King’s College London BHF Centre, The Rayne Institute, St Thomas’ Hospital, Westminster Bridge Road, London SE1 7EH, UK

**Keywords:** High-sensitivity cardiac Troponin T, Acute coronary syndrome, Rule-in/rule-out algorithm

## Abstract

**Aims:**

To evaluate the clinical feasibility of implementing the 2020 ESC 0/1 hr algorithm for rapid rule-out/rule-in of acute coronary syndrome (ACS).

**Methods and results:**

Data were collected retrospectively from 5496 patients in 2020 and 7363 patients in 2021 who received cardiac troponin measurements through the ACS algorithm in acute care settings within a large tertiary cardiac centre in the United Kingdom. This period overlapped the introduction of the 2020 ESC 0/1 hr algorithm. After exclusion of haemolysis, 1905 patients underwent repeat troponin measurement within the study period in 2020 and 2658 in 2021. Median time to repeat was significantly reduced from 3 h 14 min for intermediate low risk patients (5–12 ng/L) in 2020 to 1 h 22 min in 2021, and from 3 h 30 min to 1 h 59 min in intermediate high-risk patients (12–51 ng/L). Less than 15% of patients requiring repeat testing had dynamic changes in troponin of sufficient magnitude to change their initial risk category. Of all patients, 58.1% of patients in 2020 were ultimately classified as ‘low risk’, 19.2% deemed ‘ACS likely’, and 22.7% as ‘ACS possible’, with similar distributions in 2021.

**Conclusion:**

Whilst an efficient algorithm, our study demonstrates multi-faceted, practical limitations of achieving the 1 h target for the triage of patients with suspected ACS. Despite challenges predominantly of logistic nature, the algorithm enables rapid, streamlined, and efficient triage of large patient cohorts. Further work is required to streamline this process and achieve the targeted 1 h repeat in a resource-constrained healthcare environment, which would invariably require second blood draw before the result of first, as recommended by the ESC.

## Graphical Abstract

**Graphical Abstract:**
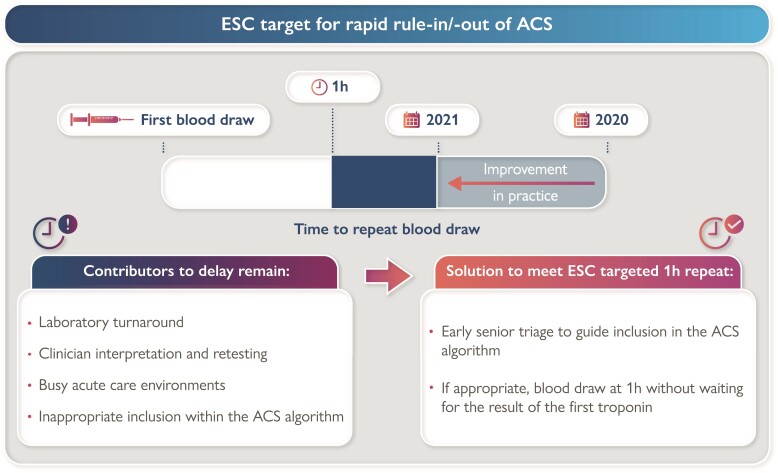

## Introduction

Acute chest pain represents one of the commonest presentations to emergency departments (EDs) in the UK, with acute coronary syndrome (ACS) representing a common and important differential diagnosis.^1^ Indeed, this is an area of constant evolution in clinical practice: the European Society of Cardiology (ESC) published new guidelines in 2020 for the management of ACS in patients presenting without ST-segment elevation,^2^ and the 4th Universal Definition of Myocardial Infarction was published in 2018.^3^ Owing to the high sensitivity and diagnostic accuracy of newer troponin assays,^4^ many centres now utilize rapid ‘rule-in’ and ‘rule-out’ algorithms with shortened time between first and second blood draws for troponin measurement, to reduce delays in diagnosis and discharge/treatment.

These guidelines rule-out patients based on either a first troponin value significantly below the 99th centile or a repeat measurement at 1 h without dynamic changes between first and second blood draw; and rule-in patients with a first troponin measurement significantly above the 99th centile or a significant change in troponin concentration at 1 h. The ESC recommends using this 0/1 h algorithm^2^ which has shown to be superior to the 0 h/3 h algorithm in terms of safety and efficacy.^5–7^

In 2015, we introduced of the 0 h rule-in/rule-out component of the ESC algorithm at St Thomas’ Hospital—an acute hospital based in central London and home to a tertiary cardiac unit. We showed this implementation enabled rapid triage of approximately half of presenting patients and was associated with more rapid repeat testing in intermediate-risk patients.^8^ This internal guideline was updated to incorporate the changes to the 0 h/1 h algorithm in August 2020. This present study aims to evaluate the clinical implementation of these protocols (during 2020 and 2021) to establish clinical feasibility in the resource-constrained environment of public healthcare.

## Methods

### Study design

This was a retrospective observational study where data was collected for all high-sensitivity cardiac troponin T (hs-cTnT) assays performed on patients presenting to the emergency department (ED) or Ambulatory Emergency Care (AEC) at St Thomas’ Hospital, between February and November for 2020 and 2021. The 0/1 h algorithm was implemented in August 2020, and significant work was carried out to encourage adoption between November 2020 and February 2021 including regular staff training and posters.

### Troponin assay

The Roche cobas 8000 platform was utilized for quantifying hs-cTnT: 99th percentile of a healthy reference population 14 ng/L; limit of blank at 2.53 ng/L; limit of detection at 3.16 ng/L; limit of quantification 3.82 ng/L; and measuring range 3–10 000 ng/L. Precision: repeatability = coefficient of variation (CV) 2.0% at 27.9 ng/L and CV 1.1% at 2084 ng/L; and intermediate = CV 2.7% at 27.9 ng/L and CV 2.1% at 2084 ng/L.

### Data sources

Patients receiving troponin measurements in ED or AEC throughout the study period were identified through an e-Audit function in ‘Symphony’, the software used within our ED, and their presenting complaint was logged for analysis. All troponin measurements for these patients were obtained upon request from ViaPath at St Thomas’ Hospital. A repeat hs-cTnT measurement was defined as any subsequent measurement on the same patient within 24 h. Patients’ demographic information was obtained from our ‘Electronic Patient Record’ or discharge records. Discharge diagnoses were recorded according to the 10th International Statistical Classification of Diseases and Related Health Problems (ICD-10) listed on our clinical notes system ‘e-Noting’ as per the discharging clinician, and were categorised into diagnostic groups. Hospital numbers unique to each patient were used for data linkage.

### The 0/1 h algorithm

**Figure 1:**
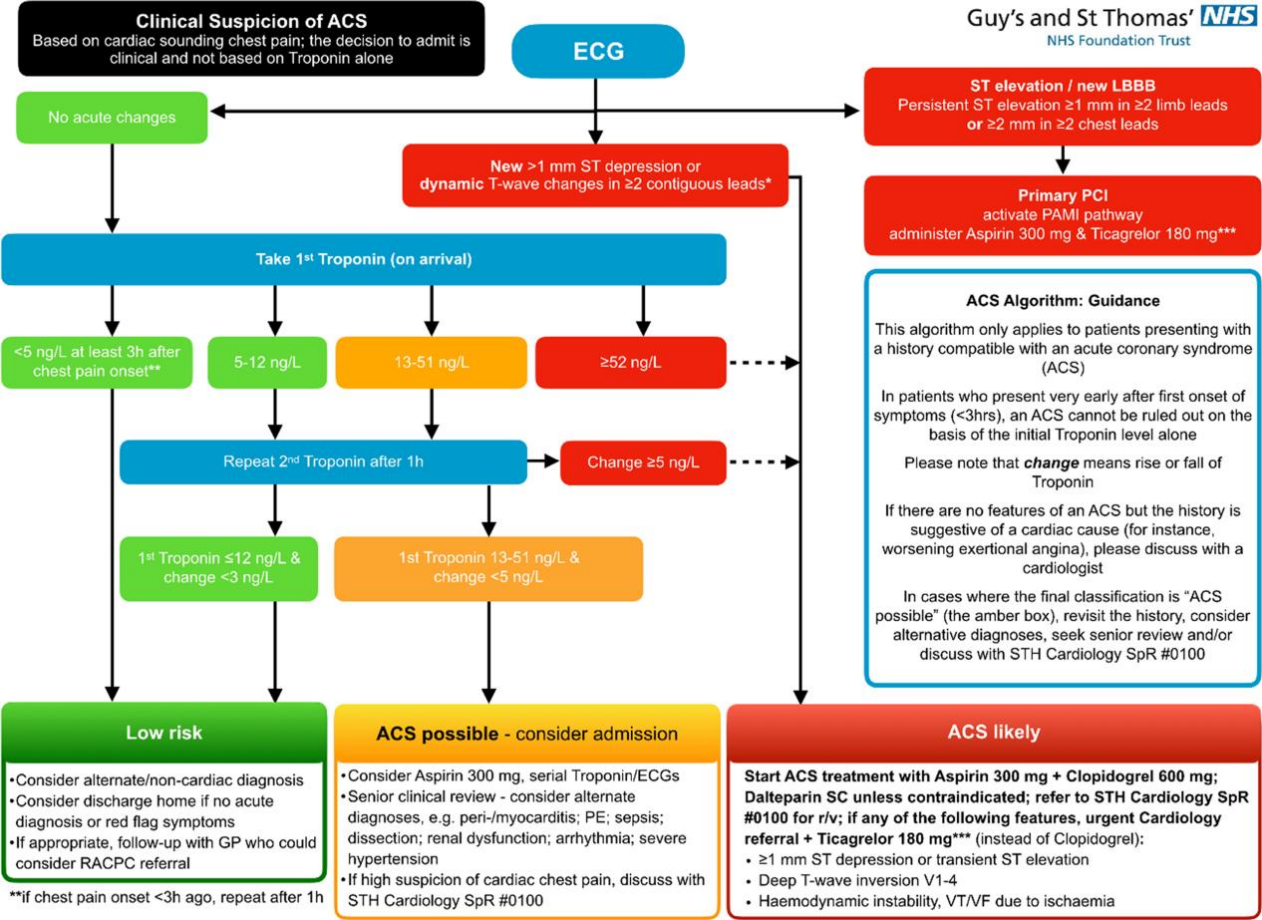
GSTT 0/1 h ACS algorithm GSTT guidelines for the management of the clinical suspicion of ACS. Patients triaged into the algorithm enter the troponin risk stratification stage if there are no acute ischaemic changes on ECG.

The 0/1 h algorithm for the diagnostic management of suspected non-ST ACS can be seen in *Figure 1*. hs-cTnT is measured on arrival to ED for patients with a history suggestive of ACS, and an electrocardiogram (ECG) without persistent ST-segment elevation.

For the purpose of this analysis, the categories based on initial troponin measurement were defined as: ‘low’ if initial hs-cTnT <5 ng/L; ‘intermediate low’ if hs-cTnT 5–12 ng/L; ‘intermediate high’ if initial hs-cTnT 13–51 ng/L; and ‘high’ if initial hs-cTnT ≥52 ng/L.

Based on these categories (*Figure 1*), patients can be immediately risk stratified to ‘low risk’ for ACS with a hs-cTnT on presentation of <5 ng/L if chest pain onset was at least 3 h prior, or ‘ACS likely’ if initial hs-cTnT ≥52 ng/L. Where initial troponin is 5–51 ng/L and patients are in the ‘intermediate low’ or ‘intermediate high’ categories, a repeat is advised after 1 h to establish dynamic change (Δhs-cTnT) to further risk stratify into ‘low risk’, ‘ACS possible’, or ‘ACS likely’ as shown in *Figure 1*.

Patients were excluded from analysis if the first sample haemolysed. Those hs-cTnT measurements returned below the limit of blank (<3 ng/L) were all ascribed a value of 2.99 ng/lL to allow for data analysis.

### Data analysis

Continuous variables were assessed for normality using Shapiro–Wilk test. Non-parametric data are expressed as median (interquartile range; IQR). Statistical significance is established with Mann–Witney *U* test or by using two-way ANOVA with Tukey’s *post hoc* for data based on two variables. Data analysis was carried out using GraphPad Prism (version 9.0 for Windows, GraphPad Software, San Diego, California USA).

## Results

During the study period, initial hs-cTnT measurements were taken from 5496 patients in 2020 and 7363 in 2021 (see Supplementary material online, *Figure S1*). Of those patients, 1905 vs. 2658 received repeat measurement in 2020 and 2021, respectively. The breakdown of the presenting complaints assigned at triage within 2020 can be seen in *Table 1*, with chest pain accounting for 56.9% of presenting complaints in all the tests performed. The next commonest single presentation was shortness of breath, at 13.6%.

**Table 1:** Summary of the presenting complaints of all patients with hs-cTnT measure in acute care settings (in descending order of prevalence)

Presenting complaint	*N*	%
Chest pain	2731	56.9%
Other	1044	21.7%
Shortness of breath	652	13.6%
Abdominal pain	165	3.4%
Collapsed adult	131	2.7%
Falls	51	1.1%
Back pain	29	0.6%
Overall	4803	100%

This table represents all patients by presenting complaint in descending order of prevalence who came to the emergency department or ambulatory emergency care and underwent a hs-cTnT measurement during the study period. *N* is number of patients displayed with whole number percentages (%).

### The 0 h troponin measurements

**Figure 2:**
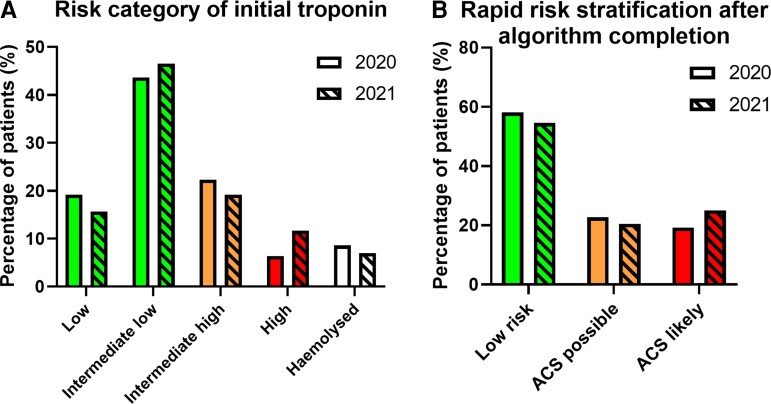
Risk categorization after troponin measurement. (*A*) Distribution of patients after the initial hs-cTnT measurement: risk categories following the 0 h troponin quantification from patients presenting to the emergency department or ambulatory emergency care, defined as: Low <5 ng/L; Intermediate low 5–12 ng/L; Intermediate high 13–51 ng/L; High ≥52 ng/L. Proportion of initial tests haemolysed shown. (*B*) Rapid risk stratification after repeat troponin measurement into ‘Low risk’, ‘ACS possible’ or ‘ACS likely’ as per *Figure 1*. Bar colours correspond to colour coding of risk as depicted in *Figure 1* with 2020 clear and 2021 hatched.

Based on the initial troponin measurements (*Figure 2A*), the majority of patients were categorised as ‘intermediate low risk’ in 2020 and 2021 (43.6 and 46.5%, respectively). ‘Intermediate high-risk’ constituted 22.3% of patients in 2020 and 19.2% in 2021, with ‘low risk’ constituting 19.2% in 2020 and 15.7% in 2021. Finally, 6.4% of patients in 2020 and 11.7% of patients in 2021 were classed as ‘high risk’. The proportion of first tests that haemolysed is also shown (8.6 and 7.0%, respectively).

### Rapid risk stratification

The repeat hs-cTnT measurements completed risk stratification as per the ESC 0/1 h algorithm (*Figure 2B*). The majority of patients were eventually classified as ‘low risk for ACS’, with 58.1% in 2020 and 54.5% in 2021 either receiving an initial hs-cTnT result of <5 ng/L (more than 3 h after the onset of chest pain), or intermediate low initial values (5–12 ng/L) with Δhs-cTnT <3 ng/L. For ‘ACS likely’, this was 19.2% and 25.0% respectively; these included patients with initial hs-cTnT measurements classified as ‘high’ risk, or under in ‘intermediate low’ or ‘intermediate high’ categories with dynamically changing hs-cTnT. The remaining patients, who were classified as intermediate high without a subsequent dynamic change (<5 ng/L), or intermediate low with a borderline dynamic change (≥3 but <5 ng/L) were classed as ‘ACS possible’ (22.7% for 2020 and 20.5% for 2021).

### Repeat troponin measurements

According to the new 0/1 h algorithm (*Figure 1*), patients with intermediate low or intermediate high initial hs-cTnT concentrations should undergo a 1 h repeat troponin measurement. As shown in *Table 2*, in 2020 only 789 patients (33.0%) in the ‘intermediate low’ category underwent repeat troponin measurement, whereas for ‘intermediate high’, this was 806 patients (65.9%). For 2021, this was similar at 1309 patients (38.3%) and 996 patients (70.3%) in each category, respectively. Patients in the ‘high’ risk category with initial concentrations ≥52 ng/L, that do not require repeat measurements within the ACS algorithm, underwent repeats in 74.8% of cases (264 patients) in 2020, which reduced to 36.6% (314 patients) in 2021. Interestingly, patients in the ‘low’ risk category still underwent repeats in 4.4% (46 patients) and 3.4% (39 patients) in 2020 and 2021, respectively.

**Table 2:** Patients receiving repeat hs-cTnT measurements according to initial risk category

Initial troponin risk category	Total number of patients		Patients having repeat test within 24 h		Of the repeats, number of patients where delta ≥3 and <5 ng/L		Of the repeats, number of patients where delta ≥5 ng/L	
	2020	2021	2020	2021	2020	2021	2020	2021
Low	1054	1153	46 (4.4%)	39 (3.4%)	0 (0.0%)	2 (5.1%)	0 (0.0%)	2 (5.1%)
Intermediate Low	2394	3422	789 (33.0%)	1309 (38.3%)	45 (5.7%)	52 (4.0%)	22 (2.8%)	31 (2.4%)
Intermediate High	1223	1416	806 (65.9%)	996 (70.3%)	150 (18.6%)	151 (15.2%)	185 (23.0%)	185 (18.6%)
High	353	858	264 (74.8%)	314 (36.6%)	18 (6.8%)	30 (9.6%)	198 (75.0%)	210 (66.9%)
Haemolysed	472	514	344 (72.9%)	379 (73.7%)	—	—	—	—

Data displayed as *N* (%) where *N* = number of patients and % = percentage of patients within each category. Percentage of patients who had repeats is expressed as a proportion of all patients within each initial risk category, whereas the percentage within each delta category is expressed as a proportion of those who received repeats.

Of the patients with ‘intermediate low’ initial hs-cTnT measurements who received repeat troponin measurement, 5.7% (45 patients) resulted in a 1 h hs-cTnT change (Δhs-cTnT) of ≥3 & <5 ng/L and a further 2.8% (22 patients) ≥5 ng/L. For 2021, this was 4.0% (52 patients) and 2.4% (31 patients), respectively. As per *Figure 1*, all those with delta ≥3 ng/L remained within the algorithm as ‘ACS possible’. A Δhs-cTnT ≥5 ng/L was required to rule-in patients within the intermediate high category. This was the case for 23.0% of patients (185 patients) in 2020 and 18.6% (185 patients) in 2021. Patients with ‘high’ initial hs-cTnT levels (≥52 ng/L) had a high proportion of dynamic change, with a delta change of ≥5 ng/L in 75.0% of cases (198 patients) in 2020 and 66.9% (210 patients) in 2021.

Where the first blood test haemolysed (8.6% in 2020 and 7.0% in 2021, *Figure 2A*), 72.9% (344 patients) received a repeat measurement in 2020, and 73.7% (379 patients) in 2021; the risk category of patients requiring repeat measurements for haemolysis can be seen in Supplementary material online, *Table S1*.

**Figure 3:**
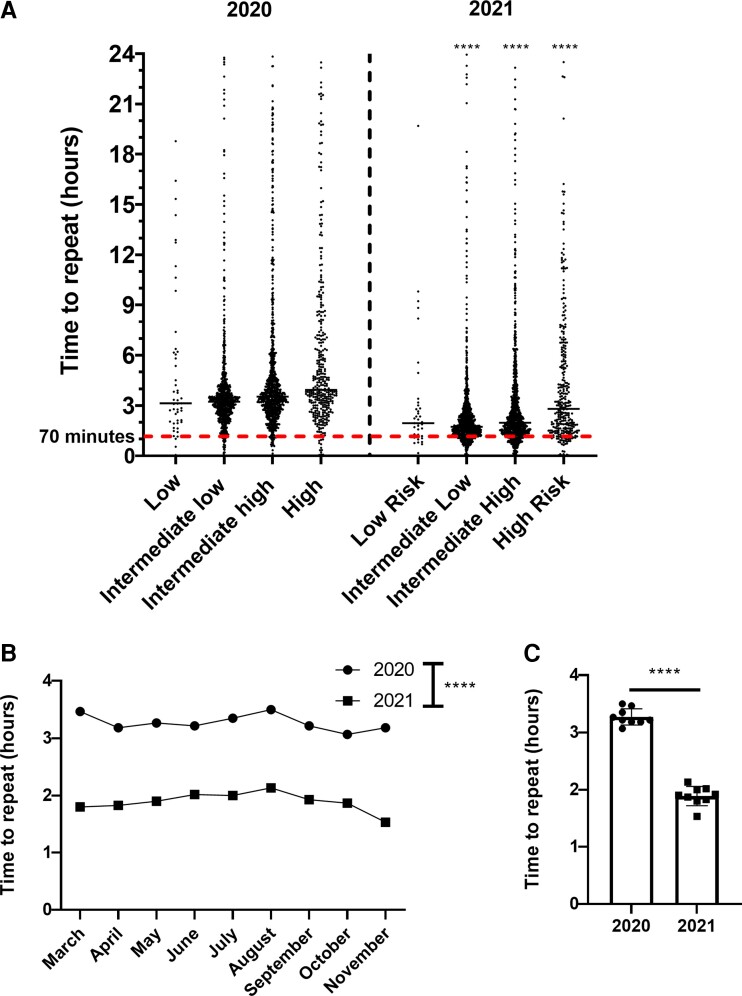
Time to repeat troponin measurement in 2020 vs. 2021. (*A*) Spread of data for time to repeat troponin stratified by initial troponin measurement. (*B*) Time to repeat troponin stratified by month. (*C*) Overall time to repeat troponin measure, where time is are averaged by month. Time (hours) on the *y* axis, with the median and spread of individual datapoints displayed as appropriate. Data not normally distributed, determined using the Shapiro–Wilk test. Statistical analysis was performed with two-way ANOVA for (*A* and *B*) with Tukey’s *post hoc* and significance for 2021 vs. 2020 is displayed on the graph for each risk category for (*A*). For (*C*), significance determined using Mann–Whitney *U* test.

As per the ESC guidelines and adapted in the GSTT 0/1h-algorithm, the recommended time to repeat testing is 1 h following the initial hs-cTnT measurement. *Figure 3A* shows the distribution of time to repeat measurements for each initial risk category, with patients defined as having repeat measurements if taken within 24 h from an initial measurement in ED or AEC, to any subsequent location within the hospital. Median time taken for ‘Intermediate Low’ significantly reduced from 3 h 14 min (IQR: 2 h 30 min to 3 h 54 min) in 2020 to 1 h 44 min in 2021 (IQR: 1 h 17 min to 2 h 28 min). Similarly, for ‘Intermediate High’, time taken significantly reduced from 3 h 30 min (IQR: 2 h 41 min to 4  35 min) to 1 h 59 min (IQR: 1 h 24 min to 3 h 26 min). For ‘Intermediate Low’ and ‘Intermediate High’, the percentage of tests taken within 1 h ( ± 10 min) from the initial measurement (shown as below the red dotted line) were 4.31 and 3.88%, respectively, in 2020, which improved to 18.0 and 16.1%, respectively, in 2021. Time to repeat for haemolysed samples was 1 h 31 min (IQR: 1 h 2 min to 2 h 15 min) in 2020 and 1 h 34 min (IQR: 1 h 7 min to 2 h 13 min) in 2021 (data not shown). Overall, the time to repeat for all categories combined did not vary significantly according to month and was significantly reduced from 3 h 13 min to 1 h 54 min for all tests overall (*Figure 3B* and *C*).

### Discharge diagnosis stratified by ACS inclusion status

**Figure 4:**
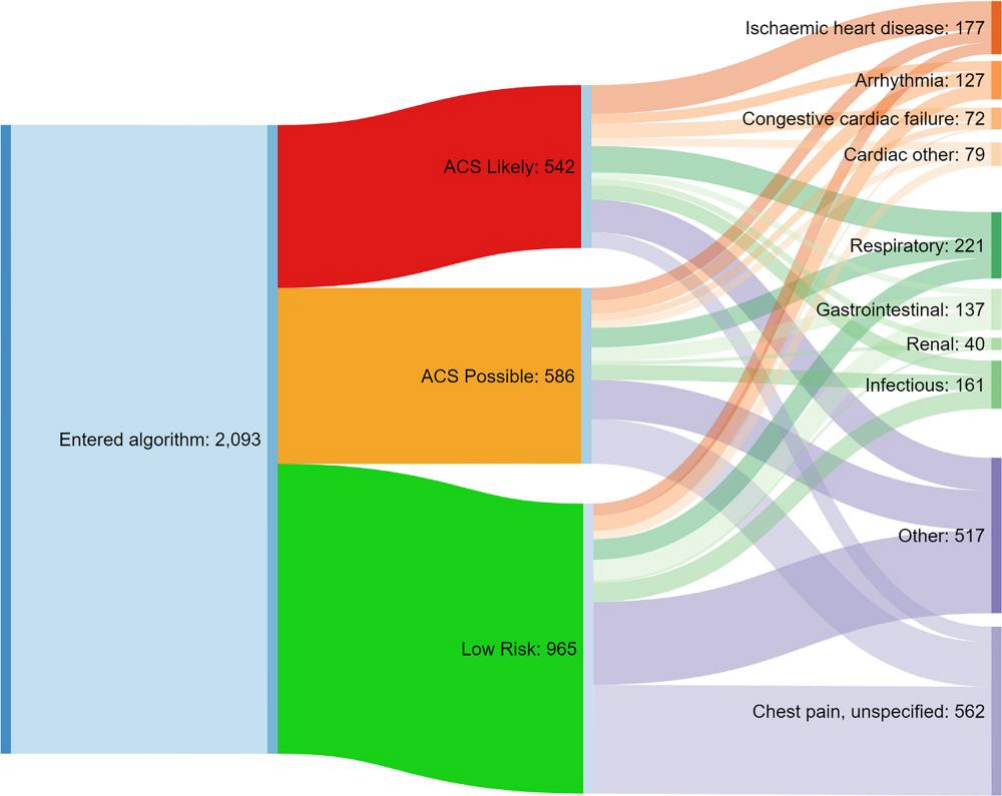
Schematic representation of triage by troponin and ultimate discharge diagnosis. Shown is the number of patients entering the ACS algorithm (left), the rapid risk stratification performed (middle), and discharge diagnoses (right). Data summarized from discharge diagnoses in Supplementary material online, *Table S1*. ‘Respiratory’ includes ‘Pulmonary embolism’, ‘Obstructive airway disease’, and ‘Respiratory other’. ‘Infectious’ includes the ‘Infectious’ category in *Table 4* and ‘COVID’. ‘Other’ includes the ‘Other’ category in Supplementary material online, *Table S1* and ‘Musculoskeletal’.

Of all discharge diagnoses, the largest single diagnostic code in patients ruled-in as per the algorithm in 2020 was ischaemic heart disease, accounting for 17.7% of all patients in this category (see Supplementary material online, *Table S2*). The largest proportion of patients deemed ‘Low Risk for ACS’ ultimately received a diagnosis of ‘unspecified chest pain’ (37.5%). Within the ‘ACS Possible’ category, 25.3% of patients were classified as ‘unspecified chest pain’. This data are visually represented in *Figure 4*. Of patients who received discharge diagnoses within the ACS algorithm, COVID represented the largest cause of in-patient mortality at 25% (see Supplementary material online, *Table S2*). A breakdown of the ICD-10 discharge diagnoses categorised within ischaemic heart disease is shown within Supplementary material online, *Table S3*, with angina the commonest overall, with the total for all angina ICD-10 codes consisting of 82 patients and 37.4% of all patients categorised as ischaemic heart disease. Acute myocardial infarction (AMI) was by far the commonest within the ‘ACS Likely’ category, with all ICD-10 codes for AMI consisting of 53 patients and 55.2% of all patients withing this category (see Supplementary material online, *Table S3*).

## Discussion

Acute coronary syndrome represents an important differential diagnosis of acute chest pain in patients presenting to acute care settings, and robust algorithms based on high-sensitivity troponin assays aid in its prompt and accurate recognition. The ESC published a refined 0/1 h hs-cTnT algorithm in 2020,^2^ which was implemented at St Thomas’ Hospital, a large tertiary centre in London, UK. We present here findings from 12 859 patients entering this algorithm in acute care settings from 2020 to 2021 to investigate feasibility of its adoption in a real-world setting.

Only about one quarter of patients could be adequately risk stratified based on the initial hs-cTnT measurement alone, i.e. those in the high risk (≥52 ng/L) and low risk (<5 ng/L) categories (*Figure 2*). Whilst rate of haemolysis matched that in published literature, the remaining patients had either intermediate low or intermediate high measurements that thus required repeats for risk stratification. Based on the 0/1 h algorithm, 58.1% of all patients in 2020 and 54.5% in 2021 were ultimately deemed low risk—an improvement in efficacy (from 40.4%) over the initial implementation of a similar rapid rule-out algorithm, which incorporated the 0 h-measurement only, in 2016.^8^ We focussed on intermediate low and high measurements for the purpose of this analysis; however, it is interesting that initial measurements classed as high risk did not have repeats in all cases (74.8% for 2020 and 36.6% for 2021). These percentages might be expected to be higher to rule-out chronic myocardial injury; however, ‘historical’ baselines may be present. Whilst the reason for the drop in repeats in 2021 is not clear, this may reflect increased prevalence of COVID and a greater appreciation of COVID myocarditis during this period.^9^

Furthermore, following implementation of the 0/1 hr hs-cTnT algorithm, only 22.7% of patients in 2020 and 20.5% in 2021 were deemed ‘ACS possible’ and required further observation vs. 51.9% in our former study.^8^ Finally, between one fifth and one quarter of patients were classified as ‘ACS likely’, whereas only 7.6% of patients were ruled-in in the previous 2015 algorithm based on the first measurement alone. This direct comparison within the same tertiary centre demonstrates that an algorithm based on repeat measurements of cardiac troponin enables more rapid triage to either low or high risk—enabling a focus on patients requiring urgent attention.

As a limiting factor, of the patients requiring repeat testing, only 33.0% of those in the ‘intermediate low risk’ and 65.9% in the ‘intermediate high-risk’ categories underwent repeat testing in 2020. This figure was largely similar in 2021 at 38.3 and 70.3%, respectively. Furthermore, the median time to repeat for all tests was longer than the targeted 1 h. While we demonstrated a significant improvement in time to repeat testing across the study period, this remains below the guideline mandated timeframes. Following discussions with clinicians in the emergency, cardiology and biochemistry departments, the following contributing factors were identified:

- The observation period analysed spanned the introduction of the 0/1 h algorithm, and thus will require time for adequate adoption. Further education and new channels for messaging (posters, screensavers, repeat communications and education) were identified as areas requiring further attention.
- Not all patients with troponin measurements are appropriate for introduction into the algorithm in the first place. Owing to logistical reasons, most blood draws are performed prior to clinical assessment by a physician—a significant proportion of patients undergo hs-cTnT testing unnecessarily and are later not subject to repeat testing requirements as the clinical picture is not felt to be consistent with ACS. This is reflective of chest pain being the presenting complaint in only 56.9% patients in 2020.
- Practical limitations: our laboratory guarantees a turnaround-time of 60 min for 80% of hs-cTnT tests received from the ED. There is, however, additional time required for (i) the physical blood draw, (ii) transfer to the lab, and (iii) reporting of the result—the latter particularly affects the service out-of-hours, as a trained biochemical scientist must sign-off on abnormal results which might further extend the time to result.
- The UK National Health System is subject to a performance target of a maximum length-of-stay of 4 h in the ED. Even with rapid triage and blood draw, initial troponin measurements may be received back within 60–90 min. With average workload, patients may be seen at ∼90 min by a clinician. Decision to repeat the blood draw may therefore occur at ∼120 min and the result from the second test would not be received for >180 min. Given the need to maintain department flow and achieved 4 h timepoints, patients are frequently moved to assessment units or the admission ward whilst awaiting further investigation, further impacting timely repeat testing and action upon results.
- Importantly, whilst the percentage of patients who strictly completed the 0/1 h algorithm in the required timeframes is low, the impact on service efficacy is felt to be greater than on safety. Whilst the ESC pathways have been validated to a time-to-repeat result in 1 h, a delay in repeat measurements is more likely to result in positive troponin deltas which are not due to AMI: as more time elapses, the delta is likely to change to a greater extent and no longer fit in with the predetermined parameters for the 0/1 h algorithm.

When considering the aforediscussion points, achieving a 1 h ( ± 10 min) repeat in a real-world setting is technically extremely difficult. Within the new ESC 2020 guidelines, the recommendation is that blood samples should be obtained at 0 and 1 h “irrespective of other clinical details and pending results”.^2^ This practice is not part of our current algorithm at GSTT and is likely a prerequisite to properly conduct the algorithm to further streamline the service. However, this requires further cost-benefit discussion within the ED, subjects many more patients to additional blood draws and could have significant knock-on effects on crowding. Furthermore, it is contrary to accepted dogma, which stipulates that an assessment of the pre-test probability of disease precedes a diagnostic test. This could be seen as particularly troublesome if results from the RAPID-TnT trial are considered, where a change in practice was observed when hs-cTnT concentrations ≤29 ng/L were reported to enable a 0/1 h hs-cTnT protocol.^10^ The authors observed an increase in invasive coronary angiography amongst patients with hs-cTnT concentrations between 5–29 ng/L, whilst also demonstrating an associated increase in death or MI in this subgroup. The concern being that a change in practice (due to ‘new’ biomarker information available) may have been associated with an increased (iatrogenic) risk to patients in lower risk categories. Thus, triage from an experienced clinician within acute care settings would be required to assess the appropriateness of patients entering the ACS algorithm, and consequently being subjected to blood draws at 0 and 1 h irrespective of the pending result, as recommended by the ESC.

There are several limitations to a study of this kind. First, whilst a single-centre observational study enabled for a paired comparison with our previous data, inherent biases and confounders in clinical practice within the organisation influence the dataset obtained. The classification of any subsequent hs-cTnT test within 24 h of the first as a repeat blood draw for quantification of the delta might have captured some patients who developed new symptoms in hospital, rather than representing an intentional repeat blood draw—whilst unlikely to affect a large number, this cannot be excluded within this data set. In addition, *post hoc* diagnostic categorization based on the 4th UDMI in this large retrospective analysis was not possible since our diagnostic coding did not enable differentiation between pathological aetiologies based on single discharge diagnosis alone. Therefore, it was not possible to further explore each case in detail to precisely determine the nature of myocardial injury. Furthermore, the ICD-10 discharge diagnoses were at the discretion of the treating clinician and did not undergo *post hoc* adjudication.

## Conclusion

In summary, our study demonstrates multi-faceted, practical limitations of achieving the 1 h target in an established risk stratification protocol for the triage of patients with suspected ACS. Despite these mostly logistical challenges, the algorithm enables a rapid, streamlined, and efficient triage of a large cohort of patients. Further work is required to streamline this process to achieve the targeted 1 h repeat and may require blood to be drawn at presentation and 1 h for every patient, if applicable to enter the algorithm, irrespective of initial results or pre-test likelihood, as recommended by the ESC.

## Supplementary Material

oeac048_Supplementary_Data

## Data Availability

Available on request.

## Lead author biography

Liam Couch graduated from Imperial College School of Medicine in 2020, during which time he completed an intercalated BSc in Cardiovascular Sciences and a PhD in Cardiac Pharmacology with Professor Sian Harding. During his PhD, he investigated the roles of circulating biomarker microRNAs in the pathophysiology of Takotsubo syndrome. He is currently in an academic junior doctor role in Cardiology at St Thomas’ Hospital, London, where he is undertaking biomarker research with Professor Michael Marber. He has secured an Academic Clinical Fellowship with Oxford University, which involves run-through training in Cardiology.
