# Supplementary material for: Rapid risk stratification of acute coronary syndrome: adoption of an adapted European Society of Cardiology 0/1-hour troponin algorithm in a real-world setting
Source: Eur Heart J Open. 2022 Jul 29;2(4):oeac048. doi: 10.1093/ehjopen/oeac048 (PMC9404254; doi:10.1093/ehjopen/oeac048)
Supplement: oeac048_Supplementary_Data [file oeac048_supplementary_data.docx]

**Supplementary Material**

**Rapid risk stratification of acute coronary syndrome: adoption of an adapted European Society of Cardiology 0/1-hour troponin algorithm in a real-world setting**

Liam S Couch MBBS PhD^1^, Aish Sinha MBBS^1^, Roshan Navin MBBS^2^, Laura Hunter MBBS^3^, Divaka Perera MBBS PhD^1^, Michael S Marber MBBS PhD^1^, Thomas E Kaier MD MBA PhD^1^

^1^ King’s College London BHF Centre, The Rayne Institute, St Thomas’ Hospital, London, UK

^2^ St Thomas’ Hospital, Acute Medicine Department, Westminster Bridge Rd, London, UK

^3^ St Thomas’ Hospital, Emergency Department, Westminster Bridge Rd, London, UK

Corresponding author:

Dr Thomas E Kaier, The Rayne Institute, 4th Floor Lambeth Wing, St Thomas’ Hospital, Westminster Bridge Road, London SE1 7EH, UK

Tel: +44-(0)20-7188 1048.

Email: Thomas.kaier@kcl.ac.uk

**Supplementary materials:**

**Supplementary Figure 1.** Total number of troponin measurements performed throughout the study period.

Total troponin measurements N = 5496

Total troponin measurements N = 7363

2020

2021

Successful first measurement N = 5024

Haemolysed first measurement N = 472

Successful first measurement N = 5634

Haemolysed first measurement N = 443

Repeat measurement N = 1905

Repeat measurement N = 2658

Haemolysed first measurements were excluded for data analysis purposes. N = number of patients.

**Supplementary Table 1. Troponin risk category in repeat after haemolysis**

| **Follow-up troponin risk category** | **Total number of patients** | |
| --- | --- | --- |
|  | **2020** | **2021** |
| **Low** | 41 (12%) | 24 (6%) |
| **Intermediate Low** | 119 (35%) | 152 (40%) |
| **Intermediate High** | 116 (34%) | 120 (32%) |
| **High** | 50 (15%) | 58 (15%) |
| **Haemolysed** | 18 (5%) | 25 (7%) |

Of samples that were haemolysed on first measurement, initial risk category of repeat troponin measurement is shown as N (%), where N = number of patients and the % = percentage of repeat haemolysed samples.

**Supplementary Table 2.** Discharge diagnosis of patients entering the 0/1hr algorithm in 2020

| **Discharge diagnosis** | **ACS Likely** | | **ACS Possible** | | **Low Risk** | | **Overall Inpatient Mortality** | |
| --- | --- | --- | --- | --- | --- | --- | --- | --- |
|  | **N** | **%** | **N** | **%** | **N** | **%** | **N** | **%** |
| Aortic dissection | 5 | 0.9% | 0 | 0.0% | 1 | 0.1% | 0 | 0.0% |
| Arrhythmia | 33 | 6.1% | 44 | 7.5% | 50 | 5.2% | 0 | 0.0% |
| Cardiac other | 22 | 4.1% | 24 | 4.1% | 27 | 2.8% | 1 | 1.4% |
| Chest pain, unspecified | 52 | 9.6% | 148 | 25.3% | 362 | 37.5% | 0 | 0.0% |
| Congestive cardiac failure | 47 | 8.7% | 23 | 3.9% | 2 | 0.2% | 3 | 4.2% |
| COVID | 36 | 6.6% | 37 | 6.3% | 43 | 4.5% | 29 | 25.0% |
| Gastrointestinal | 21 | 3.9% | 46 | 7.8% | 70 | 7.3% | 1 | 0.7% |
| Infectious | 12 | 2.2% | 13 | 2.2% | 20 | 2.1% | 3 | 6.7% |
| Ischaemic heart disease | 96 | 17.7% | 42 | 7.2% | 39 | 4.0% | 4 | 2.3% |
| Musculoskeletal | 15 | 2.8% | 34 | 5.8% | 56 | 5.8% | 1 | 1.0% |
| Obstructive airway disease | 14 | 2.6% | 15 | 2.6% | 11 | 1.1% | 1 | 2.5% |
| Other | 94 | 17.3% | 98 | 16.7% | 220 | 22.8% | 7 | 1.7% |
| Pulmonary embolism | 14 | 2.6% | 8 | 1.4% | 13 | 1.3% | 1 | 2.9% |
| Renal | 21 | 3.9% | 12 | 2.0% | 7 | 0.7% | 0 | 0.0% |
| Respiratory other | 60 | 11.1% | 42 | 7.2% | 44 | 4.6% | 9 | 6.2% |
| Overall | 542 | 100.0% | 586 | 100.0% | 965 | 100.0% | 60 | 2.9% |

Categorised discharge diagnoses made from ICD-10 codes. Discharge diagnoses classified as ‘Other’ included: malignant neoplasm of organs in a system not otherwise stated; haematological; autoimmune; endocrine; psychiatric; neurological; dermatology; toxicology; vascular (excluding aortic dissection); ear nose and throat; and surgical conditions. N = number of patients and % = percentage, where percentage for ‘ACS Likely’, ‘ACS Possible’ and ‘Low Risk’ are as a percentage of the total for each respectively category, and Overall Inpatient Mortality is as a percentage of total number of patients with each diagnosis.

**Supplementary Table 3.** ICD-10 discharge diagnoses for those categorised as ‘Ischaemic heart disease’

| **Diagnosis** | **All** | | **ACS Likely** | | **ACS Possible** | | **Low Risk** | |
| --- | --- | --- | --- | --- | --- | --- | --- | --- |
|  | **N** | **%** | **N** | **%** | **N** | **%** | **N** | **%** |
| I20.0: Unstable angina | 6 | 2.7% | 0 | 0.0% | 2 | 4.8% | 3 | 7.7% |
| I20.1: Angina pectoris with documented spasm | 3 | 1.4% | 1 | 1.0% | 1 | 2.4% | 1 | 2.6% |
| I20.8: Other forms of angina pectoris | 6 | 2.7% | 0 | 0.0% | 3 | 7.1% | 2 | 5.1% |
| I20.9: Angina pectoris - unspecified | 67 | 30.6% | 6 | 6.3% | 19 | 45.2% | 24 | 61.5% |
| I21.0: Acute transmural myocardial infarction of anterior wall | 9 | 4.1% | 8 | 8.3% | 1 | 2.4% | 0 | 0.0% |
| I21.1: Acute transmural myocardial infarction of inferior wall | 2 | 0.9% | 2 | 2.1% | 0 | 0.0% | 0 | 0.0% |
| I21.2: Acute transmural myocardial infarction of other sites | 2 | 0.9% | 2 | 2.1% | 0 | 0.0% | 0 | 0.0% |
| I21.3: Acute transmural myocardial infarction of unspecified site | 1 | 0.5% | 0 | 0.0% | 0 | 0.0% | 0 | 0.0% |
| I21.4: Acute subendocardial myocardial infarction | 52 | 23.7% | 40 | 41.7% | 5 | 11.9% | 1 | 2.6% |
| I21.9: Acute myocardial infarction - unspecified | 2 | 0.9% | 1 | 1.0% | 0 | 0.0% | 0 | 0.0% |
| I24.9: Acute ischaemic heart disease - unspecified | 5 | 2.3% | 4 | 4.2% | 0 | 0.0% | 0 | 0.0% |
| I25.1: Atherosclerotic heart disease | 57 | 26.0% | 31 | 32.3% | 8 | 19.0% | 6 | 15.4% |
| I25.5: Ischaemic cardiomyopathy | 3 | 1.4% | 1 | 1.0% | 1 | 2.4% | 0 | 0.0% |
| I25.9: Chronic ischaemic heart disease - unspecified | 4 | 1.8% | 0 | 0.0% | 2 | 4.8% | 2 | 5.1% |

ICD-10 discharge diagnoses shown as a breakdown of those categorised as ‘Ischaemic heart disease’. N = number of patients and % = percentage, where percentage for ‘All, ‘ACS Likely’, ‘ACS Possible’ and ‘Low Risk’ are as a percentage of the total for each respectively category.
